# Prevent and report: a qualitative inquiry of student and faculty recommendations for preventing and reporting learner mistreatment

**DOI:** 10.1080/10872981.2024.2428170

**Published:** 2024-11-17

**Authors:** Alejandra Colón-López, Ashley Parish, Anne Zinski

**Affiliations:** a Department of Medical Education, University of Alabama at Birmingham Heersink School of Medicine, Birmingham, AL, USA; b Department of Physical Therapy, University of Alabama at Birmingham School of Health Professions, Birmingham, AL, USA

**Keywords:** Mistreatment, prevention, reporting, medical students, faculty

## Abstract

Many medical schools in the United States (US) have employed policies and programming to prevent mistreatment and encourage students to report mistreatment events. Yet, there is little evidence showing a large-scale decrease in mistreatment behaviors overall, and, in many cases, mistreatment events go unreported. This study examines views from medical students and faculty for preventing mistreatment during medical training, as well as strategies for encouraging learners to report mistreatment events when they occur. We conducted in-depth interviews and focus groups with students and faculty (*n* = 25) and compared and contrasted perspectives. To prevent mistreatment, both students and faculty recommended institutional-level guidance and behavioral expectations during training, while faculty suggested educational programming focused on clarifying mistreatment definitions and cultivating awareness. To encourage reporting of mistreatment events, students and faculty emphasized: access to an array of reporting mechanisms, institutional processes for maintaining anonymity or confidentiality, and follow-up procedures to address reported mistreatment. Our results suggest that students’ and faculty’s role in medical education may shape their perceptions of strategies to prevent mistreatment. These results can inform the development and customization of interventions for preventing mistreatment and encouraging mistreatment reporting.

## Introduction

‘Behaviors that show disrespect for the dignity of others [and] unreasonably interfere with the learning process,’ also known as learner mistreatment, have been linked with multiple negative implications for students, including sub-optimal learning, poor mental health, specialty choice reconsideration, and medical school attrition [1–3]. The prevalence of learner mistreatment in allopathic (MD) medical schools has prompted ongoing monitoring of mistreatment behaviors by accrediting bodies [4,5]. Schools in the United States (US) have responded by implementing an array of policies and programming, yet mistreatment reports persist in this training environment [6–10].

According to recent literature, various prevention strategies are used in US medical schools to mitigate the prevalence of mistreatment behaviors [11]. Many employ targeted curricula with mistreatment definitions and/or case-based discussions to train learners to identify and respond to mistreatment behaviors [7,12–18]. Other schools have introduced or revised policies and guidelines for professional conduct for students and faculty [9,19–22], and launched mistreatment awareness campaigns [6]. Published scholarship documents some effectiveness of prevention-oriented programming, yet large-scale decreases in mistreatment behaviors are not well documented [20,23].

In addition to prevention, researchers have investigated reporting as a mechanism for addressing mistreatment [24–27]. Between 2019 and 2023, approximately three-quarters of graduating medical students who reported at least one personal experience with mistreatment did not report the incident to their institution [5]. Although recent studies document factors that influence medical students’ reporting decisions [27–29], these studies rarely examine mechanisms for encouraging reporting of mistreatment behaviors by both learners and faculty.

Though strategies to decrease and prevent learner mistreatment have been implemented in US medical schools, fewer studies examine how one’s role in medical education might shape opinions on preventing mistreatment and encouraging students to report mistreatment behaviors [26,27]. In this investigation, we use qualitative methods to identify students’ and faculty’s converging and contrasting views on preventing and reporting mistreatment events, and we discuss how findings may guide the development of interventions to address mistreatment in medical education.

## Methods

### Study design

In this study, we employ Constructivist Grounded Theory (CGT) methods, including iterative data gathering and coding and inductive thematic analysis [30]. This methodology supports the discovery of data-driven theories and explanations of a phenomenon [30,31]. We apply CGT methods to explore medical students’ and faculty’s converging and differing opinions on preventing mistreatment behaviors and encouraging students to report mistreatment events [32]. This approach is appropriate for this study because it examines individuals making sense of their interactions with others within the social environment.

The research team is composed of two faculties and a postdoctoral trainee. ACL has a background in Medical Sociology, and she is a postdoctoral scholar in the Department of Medical Education. AP holds a faculty appointment in the Department of Physical Therapy, School of Health Professions. AZ is the Director of Educational Research, Evaluation, and Assessment in the Department of Medical Education.

### Recruitment and sampling

We employed convenience sampling to recruit students and faculty from a public MD-granting institution in the Southeastern US. We invited prospective participants from a group of 80 faculty and 30 students who had expressed interest in discussing the learning environment during a previously administered local survey. Two students who had not participated in the survey were also enrolled after responding to an email invitation sent to students and faculty and sharing their interest in participating in this study. Participants were recruited based on interest in participation, regardless of their experience with learner mistreatment.

### Data collection

Using published multi-method guidelines, we collected participants’ responses from focus group discussions and in-depth interviews [33,34]. During recruitment, participants were asked whether they preferred to give an in-depth interview or participate in a focus group discussion. We employed both data collection strategies to allow participants to choose their preferred method of engagement. [33] Participants who chose to participate in a focus group discussion were stratified to a group session by their role in medical education: medical student or faculty.

ACL and AZ developed two parallel in-depth interview/focus group guides: one for medical students and one for faculty (See Appendix A). Both guides included common questions asking participants about their general understanding of learner mistreatment and programming for addressing mistreatment. Participants were asked to share their personal meaning of learner mistreatment along with their recommendations for preventing mistreatment behaviors and encouraging students to report mistreatment events. The discussion guide for students included additional questions regarding personal reporting experiences and related preferences.

All interviews and focus group sessions were conducted during April 2023 by an experienced qualitative researcher (ACL). All sessions were conducted via video call. The study’s purpose, ethical review information, and instructions for the video call were shared with participants prior to each session. All participants were given the option to turn their camera on or off at any time. All interviews and focus group meetings were audio recorded and stripped of identifiers during transcription. The University of Alabama at Birmingham Institutional Review Board provided ethical approval for this study.

The data that support the findings of this study are available from the corresponding author, ACL, upon reasonable request.

### Data analysis

Two experienced qualitative researchers (ACL and AP) independently read all transcripts and employed line-by-line coding [35]. During team meetings, ACL and AP discussed open codes and AZ moderated the conversation. Codes were interactively modified until the team agreed on a codebook. ACL and AP used NVivo14 to employ a consensus-based coding approach to four full transcripts chosen at random [36]. After initial comparison, ACL and AP discussed coding discrepancies, modified the employed coding scheme, and independently coded the remaining transcripts. The final Kappa coefficient was 0.82, and the coding agreement was 99.72%. During a series of analytic meetings, the research team identified final themes and sub-themes and constructed thematic categories from responses.

## Results

**Figure 1.**
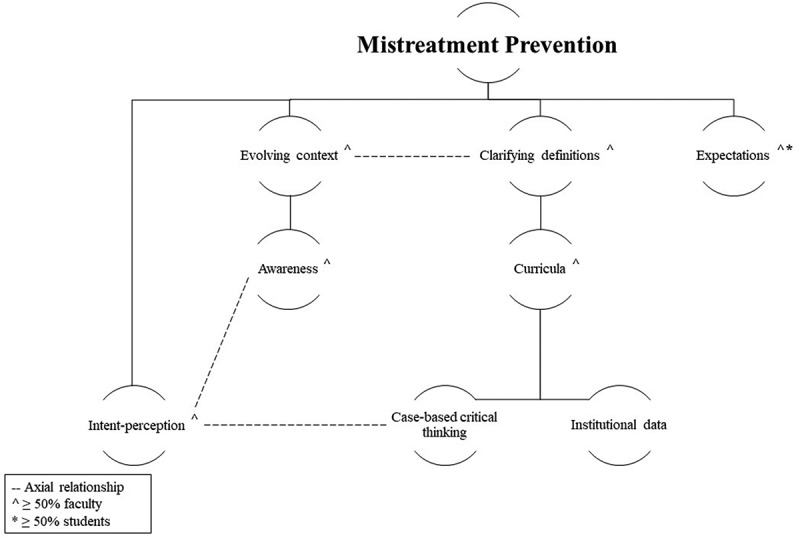
Mistreatment revention themes.

**Figure 2.**
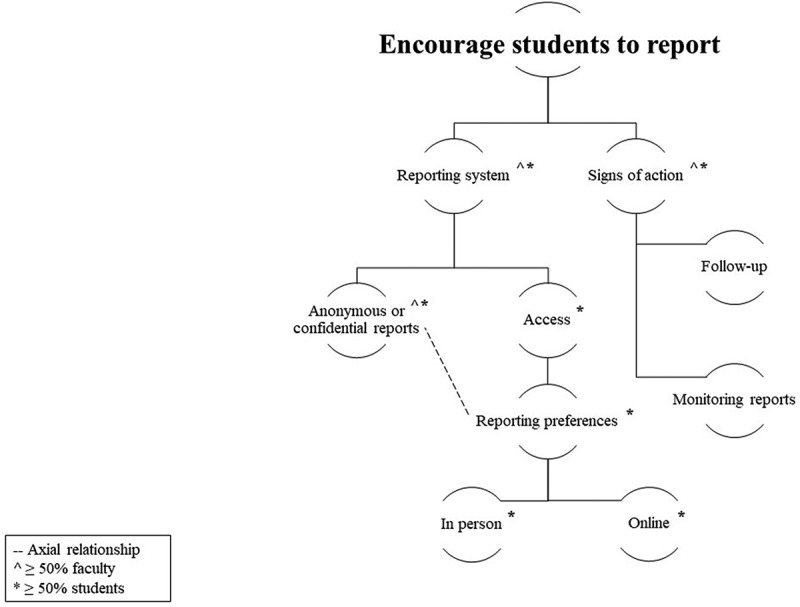
Encourage students to report themes.

Thirteen in-depth individual interviews and four focus groups with eight medical students and 17 faculties (*n* = 25) were conducted, with interviews lasting between 32 and 56 minutes and focus groups lasting 58 to 65 minutes. Participating faculty taught at some capacity at the medical school (preclinical or clinical phase), and students represented all years of undergraduate medical training. Per participant comfort, demographic information was not collected. Four themes emerged from transcripts (See Figures 1 and 2). The following sections provide descriptions of themes and subthemes, which are supported with relevant excerpts.

### Suggestions for preventing mistreatment

Most respondents, indicated that people may not be aware of what behaviors actually constitute learner mistreatment, and these individuals may demonstrate mistreatment behaviors unintentionally or misinterpret other behaviors. The majority of faculty suggested strategies for clarifying mistreatment definitions with an acknowledgement that definitions evolve (Figure 1). Both students and faculty expressed their desire for clear training expectations by role to encourage acceptable behaviors.

#### Evolving mistreatment definitions

Most faculty respondents acknowledged that the context of medical education has been evolving, and updates to standards and policies have reshaped mistreatment definitions. This observation became apparent when faculty participants stated that some behaviors that were once ‘acceptable’ as part of the training culture may no longer be suitable for medical training: ‘*Thirty years ago, people thought some things were acceptable, and for some of our faculty that was the last time they were involved with learners*.’ [F13] Because those who are less familiar with current mistreatment literature may unintentionally display mistreatment behaviors, faculty respondents believed that awareness programming on the evolution of the training environment could minimize the incidence of unintentional mistreatment behaviors: ‘*The school can raise people’s awareness and tell them, “This is not appropriate and there’s something you can do about it*.”’ [F07]

Faculty indicated that they personally struggle to identify mistreatment behaviors because they are unsure which behaviors constitute mistreatment. As some individuals may rely on intuition to interpret these interactions, these events may be misconstrued without a clear and current mistreatment definition: ‘*Students can misconstrue behaviors that make them uncomfortable as mistreatment, whether they are from faculty members, other learners or peers. People will have different definitions*..’ [F12] Therefore, the faculty in this study recommended updated curricula with clear mistreatment definitions to align mistreatment perceptions between students and faculty.

While recognizing the need for clearer and current definitions, several faculty described instances in which students and faculty could not agree on whether a behavior constituted mistreatment due to differences in points of view: ‘*It really depends on how the student perceived [the behavior.] These are bidirectional; sometimes [mistreatment] really happened, and sometimes the student overreacted*.’ [F03] Most faculty argued that students and faculty may not be aware of differing perspectives and may solely note their own perceptions on others’ behaviors. One-quarter of faculty recommended a case-based critical thinking curriculum to illuminate differing perceptions of mistreatment and communication strategies for creating shared understanding: ‘*The school could have ongoing and open conversations with faculty members and describe things that learners perceived as mistreatment*.’ [F01]

Half of the faculty in this study recognized that careful monitoring of mistreatment is a necessary step toward prevention. These participants also believed that educators should have access to institutional data to know which behaviors are prevalent and where they occur in order to address and prevent mistreatment incidents: ‘*People also liked knowing which mistreatment issues happened instead of hearing, “There are mistreatment issues. Our clerkship is poorly ranked*.”’ [F017] Some faculty respondents suggested that these data should be presented as a prelude for curriculum-based interventions.

#### Behavior and training expectations

Nearly all students and half of faculty respondents described events in which students and faculty did not know which behaviors are acceptable because they lacked clear guidelines: ‘*On day one, as a student, you don’t always get expectations and you’re sometimes afraid to ask*.’ [S04] Students and faculty who may be unaware of behavior standards may instead use their feelings (e.g., fear), intuition, or prior experience to decide which behaviors and responsibilities are acceptable. ‘*Some learners have challenges with that relationship because they don’t know what to expect from the relationship*.’ [F14] Therefore, respondents recommended publishing explicit behavioral expectations describing the student–faculty relationship throughout the medical training experience.

Both students and faculty agreed that clear expectations provide benchmarks for identifying mistreatment behaviors and minimizing disagreement on what constitutes mistreatment: ‘*Setting expectations and having clear communication with students helps prevent misalignments within these gray areas of perceived mistreatment*.’ [F04] Students inferred that training and behavioral expectations could prompt both students and teachers to demonstrate professional conduct: ‘*[The school should] also set expectations … Residents will not talk behind students’ backs or mistreat them to their face because the expectations are lined up*.’ [S03]

### Suggestions for encouraging reporting

Respondents’ recommendations for encouraging students to report mistreatment behaviors were classified into two major themes: (1) the reporting system itself, specifically features and procedures related to reports, and (2) signs of action (Figure 2). The first theme comprises attributes of a school’s reporting system that may encourage students to report mistreatment behaviors. The second theme demonstrates the relationship between evidence of prior action and students’ reporting decisions.

#### The reporting system features and procedures

Nearly two-thirds of participants in this study argued that students may hesitate to report mistreatment behaviors if they anticipate that repercussions from reporting may affect their academic standing or training experience. As students considered whether to report a potential mistreatment event, they wondered how administrators would prevent consequences that may interfere with their training or career prospects: ‘*[Students] always worry about repercussions on their grade*.’ [F13] The majority of respondents also believed students are more likely to report behaviors if they know how the reporting system will protect their identity. Thus, respondents recommended explicit measures and procedural transparency for maintaining anonymous or confidential reporting.

While discussing strategies for encouraging students to report mistreatment behaviors, half of students cited that the actual mechanism of reporting may influence reporting decisions. Several students said the school’s online reporting tool may be the most accessible, and they frequently encounter advertising campaigns for this resource: ‘*Every course director and major person in [the office of student affairs] mentioned [the online reporting tool,] and I don’t think there is a problem with advertising it*.’ [S02] Some students stated that online reporting does not limit when and from where they can submit a report: ‘*The online form is probably better … I think it’s easy because you can [submit a report] whenever you want instead of setting a meeting, and it can be anonymous*.’ [S08]

Although many students agreed that online reporting may be most accessible, they also recognized that an additional in-person reporting option may be better in some situations. In-person reporting routes allow students and administrators to exchange information that may be difficult to share through online reporting: ‘*The in-person interaction might help clarify what someone said versus a misinterpretation of something that is written*.’ [S07] Students indicated that reporting choices are determined by personal preferences: ‘*If I’m experiencing mistreatment, I’m pretty quick to say, “This isn’t fair. Someone listen to me.” I’m more open*;’ [S04] and that the nature of the behavior or interaction may also influence their reporting choice: ‘*If it involves a highly sensitive topic (e.g., inappropriate interaction with a preceptor), I would feel more comfortable submitting the report online*.’ [S06] Both students and faculty indicated that providing multiple options for reporting mistreatment behaviors can support reporting overall.

#### Signs of action

According to both students and faculty, students may be more prone to report mistreatment events centrally if they have seen evidence that reports are addressed by the school. If students, however, do not witness change, they may conclude that the reporting system does not work. Some students said that they are more likely to use the school’s local reporting system for facility issues than mistreatment behaviors because they have observed previously reported problems get fixed: ‘*They’re very responsive with maintenance requests; for example, the elevator is not working, and it gets fixed the next day. I can see what happened*.’ Additional students said they do not have confidence in the school’s reporting system because they are unsure how the school addresses non-maintenance reports.

Although most students and faculty recognized that some reports require discretion, they agreed that students should be informed about how their mistreatment reports, and mistreatment reports in general, have been addressed. Some respondents said students are more likely to trust the reporting system if they are provided with real-life scenarios and aggregated data about mistreatment and addressing actions: ‘*I think the best thing is to give examples of situations that have been resolved through [the reporting system]*.’ [S03] Meanwhile, several respondents stated that those who do not receive a response after reporting may not trust the reporting system: ‘*I reported those incidents … follow-ups might have encouraged me to have more faith in the system*.’ [S07]

## Discussion

Students’ and faculty’s role in medical education may shape their understanding of the learning environment and learner mistreatment [26,37–39]. We further explored these differences to compare medical students’ and faculty’s responses during in-depth interviews and focus groups and identify recommended strategies for preventing learner mistreatment and encouraging students to report mistreatment behaviors.

Findings indicate that students’ and faculty’s views on preventing mistreatment may be influenced by role, as student and faculty participants did not entirely agree on strategies to mitigate learner mistreatment. Consistent with published literature, faculty recommended clarifying mistreatment definitions with curricular programming [13–16,40]. However, they also recommended providing up-to-date expectations to help define currently acceptable behavior in clinical training. Students also recommended clear expectations for the learning environment; however, they did not mention or share opinions on specific mistreatment curricula. Although prior findings have emphasized students’ desire for clear training expectations [41], both student and faculty participants from this study agreed that well-defined behavioral guidelines and expectations for students and teaching faculty are crucial to prevent mistreatment behaviors.

Interestingly, faculty acknowledged that the definition and scope of mistreatment could have evolved since the last time they received formal training on learner mistreatment. These respondents also inferred that some individuals could have mistreated students unintentionally, due to unfamiliarity with revised mistreatment literature [7]. Thus, faculty believed that ongoing curriculum tools should include clear mistreatment definitions and case-based critical thinking exercises [28]. Published findings from curricular interventions show that these efforts result in effective categorization of mistreatment behaviors and greater awareness of mistreatment policies [8,12–14,16,23,40]. While effective prevention of mistreatment likely requires that students and faculty agree on a mistreatment definition [40], fewer results from curricular interventions that are delivered to faculty have been published [7,14]. This study indicates that both learners and teachers should participate in interventions to openly address differing perceptions of mistreatment. Interventions to align teachers’ and learners’ understanding of mistreatment may be more effective for prevention than addressing either group alone [7].

When guidelines for professional behaviors and responsibilities in the training environment are not provided, both students and faculty in this study reported using intuition to define the appropriateness of their interactions, as well as what constitute mistreatment [26,27]. As participants inferred that mistreatment is more likely to occur, or could be perceived to occur, when there is low or little awareness of the types of behaviors that are expected in the learning environment, a shared model for expectations and benchmarks of professional conduct is essential for identifying professional and unprofessional behaviors. While several US schools have utilized the Association of American Medical Colleges (AAMC’s) *Compact Between Resident Physicians and Their Teachers* to provide guidance for medical students and faculty, additional research on how teacher-learner compacts may affect professional behavior overall, as well as mistreatment reports, is warranted [20,42]. Further examination of the language, accessibility, and utility of published compacts is also warranted.

One particularly important finding is that student participants did not suggest curricular interventions as prevention strategies, and our data did not indicate whether student participants believed that educational interventions could prevent mistreatment behaviors. While literature on curriculum-based interventions often report favorable reviews from participants [13,43], students’ views about the need for mistreatment curriculum prior to implementation are less prevalent in the mistreatment literature. Subsequent research to explore students’ attitudes and input toward the development of interventions for preventing mistreatment may be an important next step.

In this study, opinions on reporting were less disparate by role, as students and faculty shared similar recommendations to encourage students to report mistreatment. Consistent with prior scholarship, both students and faculty agreed that reporting decisions are influenced by the reporting system itself, including the mechanism for collecting mistreatment reports, as well as the visibility of the response and subsequent follow-up [27]. As in prior studies, respondents believed that students may elect not to report mistreatment events if they are unsure whether and how their school’s reporting system will address their report [5,27,44]. This finding reinforces the need for schools to provide individual responses, as available, as well as school-level data on how mistreatment reports have been addressed.

More students than faculty discussed how specific features and access points of their school’s reporting system could influence their reporting decisions. All student participants said they learned about the school’s online reporting tool through advertising campaigns and ongoing messaging from administrators and instructors, but none said they learned about other reporting options, such as in-person reporting or the University or affiliated Hospital reporting system. Prior studies document students’ reporting experiences, factors that may influence reporting decisions, and interactions with online reporting tools [10,27,29], yet very few compare students’ interactions with, and preferences for utilizing, different reporting systems, yielding this area for additional exploration.

As reported in prior scholarship [45,46], most student and faculty participants inferred that students will choose not to report mistreatment if they anticipate retaliation, and that students consider how a system protects their identity and prevents reprisal prior to reporting [27]. Both students and faculty participants in the present study agreed that advertising the mechanisms for maintaining confidentiality or anonymity can encourage students to report mistreatment. School leaders should convey how anonymous and/or confidential reporting operates, as wells as the reporting system’s strengths, limitations, and/or legal obligations [28].

Although most students indicated that online reporting may be the most convenient [10], it may not be the best reporting option for all circumstances. Students who desire immediate feedback and/or want to discuss their interaction with another person may prefer face-to-face reporting options; students who do not feel comfortable reporting in-person and/or require formal documentation may choose online reporting means. Although online tools may facilitate the reporting process [10], schools should offer and advertise both online and in-person reporting channels in order to accommodate students’ needs and preferences [28].

All respondents agreed that students may not report because they do not know whether or how the school administration addressed prior reports. Informing students about the school’s responses to mistreatment can influence their reporting decisions [44]. In addition to students, the school’s efforts to address mistreatment must also be clear to the broader medical school community to increase both students’ and faculty’s confidence in the reporting system [47]. Some students and faculty suggested sharing facts of individual reports, aggregated data of reports, and how these were addressed. Faculty also recommended establishing robust follow-up procedures once a report is submitted [48]. Future research examining students’ preferences for individual follow-up, as well as school-level reporting of how mistreatment has been addressed, may supply guidelines for effectively documenting reports.

Findings from this investigation should be viewed considering several limitations. Results were drawn from a sample of students and faculty from one institution and may not reflect the views of students and faculty from other medical schools. Our sample has a student–faculty ratio of 1:2, and we used a convenience sample of participants who had completed a prior local survey. Additionally, per participant comfort, we did not collect individual characteristics for comparison, and not all participants chose to remain off camera, so we did not document all nonverbal cues.

These findings contribute to mistreatment prevention scholarship by exploring students’ and faculty’s converging and contrasting views on mistreatment prevention, as well as strategies to encourage students to report mistreatment events. This investigation has three primary strengths. First, the sample included students with and without a history of personal mistreatment and faculty with various teaching and leadership appointments, hence, findings represent an array of diverse viewpoints. This sampling approach also allowed us to compare faculty and student input toward generating programming to prevent mistreatment. Second, we collected respondents’ views on potential strategies prior to designing or altering local mistreatment interventions, which we expect will maximize acceptability and benefit to both populations. Third, respondents were allowed to choose to participate in an individual interview or focus group session, as wells as turn their camera on or off at any time during video calls, which allowed us to maximize our recruitment and data collection efforts [33].

## Conclusion

Prior scholarship indicates that students’ and faculty’s role in medical education may shape their perceptions of the learning environment and how they perceive professional behaviors and learner mistreatment [38,49]. In this study, we highlight these perspectives to identify students’ and faculty’s converging and contrasting recommendations for preventing mistreatment behaviors and encouraging reporting of mistreatment events. Concerning prevention strategies, both students and faculty recommended explicit guidelines for aligning expectations of professional behaviors in the learning environment; only faculty suggested curricular programming to foster awareness and supply clear mistreatment definitions. To encourage mistreatment reporting, both faculty and student respondents expressed a desire for providing mistreatment reporting options and detailing procedures for maintaining student confidentiality and efforts to address mistreatment reports, which is consistent with prior recommendations [28,50]. These results will serve as a guide for the development of tailored interventions by role for preventing mistreatment and encouraging reporting in the learning environment.
